# Quantitative Proteomics Approach to Screening of Potential Diagnostic and Therapeutic Targets for Laryngeal Carcinoma

**DOI:** 10.1371/journal.pone.0090181

**Published:** 2014-02-27

**Authors:** Li Li, Zhenwei Zhang, Chengyu Wang, Lei Miao, Jianpeng Zhang, Jiasen Wang, Binghua Jiao, Shuwei Zhao

**Affiliations:** 1 Department of Otolaryngology-Head and Neck Surgery, Changzheng Hospital, Second Military Medical University, Shanghai, China; 2 Department of Biochemistry and Molecular Biology, Second Military Medical University, Shanghai, China; 3 Key Laboratory of Liver Disease, Center of Infectious Diseases, Guangzhou 458 Hospital, Guangzhou, China; 4 Department of Pharmacology, School of Pharmacy and Institute of Biomedical Sciences, Fudan University, Shanghai, China; The Walter and Eliza Hall of Medical Research, Australia

## Abstract

To discover candidate biomarkers for diagnosis and detection of human laryngeal carcinoma and explore possible mechanisms of this cancer carcinogenesis, two-dimensional strong cation-exchange/reversed-phase nano-scale liquid chromatography/mass spectrometry analysis was used to identify differentially expressed proteins between the laryngeal carcinoma tissue and the adjacent normal tissue. As a result, 281 proteins with significant difference in expression were identified, and four differential proteins, Profilin-1 (PFN1), Nucleolin (NCL), Cytosolic non-specific dipeptidase (CNDP2) and Mimecan (OGN) with different subcellular localization were selectively validated. Semiquantitative RT-PCR and Western blotting were performed to detect the expression of the four proteins employing a large collection of human laryngeal carcinoma tissues, and the results validated the differentially expressed proteins identified by the proteomics. Furthermore, we knocked down PFN1 in immortalized human laryngeal squamous cell line Hep-2 cells and then the proliferation and metastasis of these transfected cells were measured. The results showed that PFN1 silencing inhibited the proliferation and affected the migration ability of Hep-2 cells, providing some new insights into the pathogenesis of PFN1 in laryngeal carcinoma. Altogether, our present data first time show that PFN1, NCL, CNDP2 and OGN are novel potential biomarkers for diagnosis and therapeutic targets for laryngeal carcinoma, and PFN1 is involved in the metastasis of laryngeal carcinoma.

## Introduction

Laryngeal carcinoma, one of the most common types of cancer in the head and neck, accounts for 2.4% of new malignancies worldwide every year [1], [2]. This cancer is mainly squamous cell carcinoma, reflecting its origin from the squamous cells [3]. In addition, it is approved that laryngeal carcinoma may spread by direct extension to adjacent structures, and frequently distant metastasis to the lung [4], [5]. Up to now, most patients of laryngeal cancer could retain laryngeal function after the therapy if the disease was detected at an early stage. But unfortunately, the fact is that the disease is often diagnosed at advanced stages because of the lack of reliable, early diagnostic biomarkers. Therefore, identification of biomarkers for early detection and prognosis is important and may in turn lead to more effective treatments using multiplex technologies.

Proteomics, a study of the complete protein complements of the cell, is the integration of biochemical, genetics, and proteomics data in the detection of biomarkers for early detection of cancers [6]–[8]. Proteomics is currently considered to be a powerful tool for global evaluation of protein expression, and has been widely applied. It has been suggested that analysis of the cancer proteome can be beneficial to understand not only the association between protein alterations and malignancy, but also the effect of molecular intracellular mislocalization in tumour initiation [9]. Consistently, the development of increasingly high-throughput and sensitive mass spectroscopy-based proteomic techniques provides new opportunities to examine the physiology and pathophysiology of many biological samples. The two dimensional liquid chromatography tandem MS (2D LC-MS/MS) analysis is emerging as one of the more powerful quantitative proteomics methodologies in the search for tumour biomarkers [10], [11]. For instance, in previous study the authors used 2D LC-MS/MS to identify 100 differentially expressed proteins from rheumatoid arthritis patients, and concluded that up-regulation of vasculature development related proteins and down-regulation of redox-related proteins in fibroblast-like synoviocytes were predominant factors that may contribute to the pathogenesis of rheumatoid arthritis [12]. Moreover, using LC-MS/MS, Moon et al efficiently quantified the proteins of balding and non-balding dermal papilla cells (DPCs) from patients, and 128 up-regulated and 12 down-regulated proteins among 690 distinct proteins were identified in balding DPCs compared to non-balding DPCs [13].

A number of studies using proteomics based on surface-enhanced laser desorption/ionization time-of-flight MS have identified the differential serum proteins in laryngeal carcinoma, leading to discovery of potential biomarkers for diagnosis or prognosis [14], [15]. Although some proteomic studies on laryngeal carcinoma tissue have been reported [16]–[18], there are no clinically established biomarkers available for early detection and therapeutic targets of this cancer. Therefore, to obtain more information, in the present study, 2D LC-MS/MS was performed to identify the differential proteins between laryngeal carcinoma tissue and corresponding adjacent noncancerous tissue, and then the bioinformatics analyses, including gene ontology (GO) analysis, and protein network analysis of different proteins were conducted. Subsequently, values of the four differential proteins (PFN1, NCL, CNDP2 and OGN) with expressional alterations were selectively validated by semiquantitative RT-PCR and Western blotting. Furthermore, we first time show that PFN1, NCL, CNDP2 and OGN may be potential diagnostic and therapeutic targets for laryngeal carcinoma, and demonstrate that PFN1 is involved in the migration of human squamous cells.

## Methods

### Patients

Thirty-four laryngeal carcinoma tissues and corresponding adjacent noncancerous tissues were obtained from 34 patients who underwent surgical resection in Shanghai Changzheng Hospital, in accordance with approved human subject guidelines approved by the Scientific and Ethical Committee of Second Military Medical University. And an informed consent form was signed by the participants to proceed with the protocol research. All patients undergone resection and were not treated with neoadjuvant chemotherapy or radiotherapy. Two specimens were obtained from each patient, one from the centre of the tumor and the other of similar mass from remote areas (>1 cm) adjacent to the cancerous regions. All these samples were taken by experienced surgeons and examined by experienced pathologists, frozen immediately in liquid nitrogen, and then frozen at −80°C until use. The clinical details of the patients are shown in Table 1.

**Table 1:** Clinical characteristics of the patients

Characteristic	No. of patients (%)
Number of samples	N = 34
Gender	
Male	32/34(94.12)
Female	2/34(5.88)
Age (years)	
Mean	61.2±7.4
Range	38–75
Clinical stage	
I	8/34(23.53)
II	6/34(17.65)
III	11/34(32.35)
IV	9/34(26.47)
Tumor location	
Glottic	19/34(55.88)
Supraglottic	11/34(32.35)
Subglottic	2/34(5.88)
Transglottic	2/34(5.88)

### Protein sample preparation

Samples collected from ten cancer tissues and the corresponding adjacent noncancerous tissues groups were pooled, respectively. 2 mg samples were ground in liquid nitrogen. One milliliter of lysis buffer (7 M urea, 2 M thiourea, 1x Protease Inhibitor Cocktail (Roche Ltd. Basel, Switzerland)) was added to sample, followed by sonication on ice and centrifugation at 13 000 rpm for 15 min at 4°C. The supernatant was stored in small aliquots at −80°C and the protein concentration was determined using a modified Bradford method.

### 2D-LC-MS/MS

One hundred micrograms of protein were reduced with 1 mM DTT for 45 min at 60°C, and carbamidomethylated with 5 mM iodoacetamide for 45 min at room temperature in the dark. Alkylated proteins were diluted four times with deionized water, and then digested with sequencing grade modified trypsin (Promega) overnight. The protease/protein ratio was 1: 50. The resulting peptide mixture was acidified with TFA to pH = 3, and then was desalted using a 1.3 ml C18 solid phase extraction column (Sep-Pak Cartridge) (Waters Corpoation, Milford, USA). The peptides were dried using a vacuum centrifuge and then resuspended with loading buffer (5 mM Ammonium formate containing 5% acetonitrile, pH 3.0), separated and analyzed by two-dimensional (2D) strong cation-exchange (SCX)/reversed-phase (RP) nano-scale liquid chromatography/mass spectrometry (2D-nanoLC/MS). The experiments were performed on a Nano Aquity UPLC system (Waters Corporation, Milford, USA) connected to an LTQ Orbitrap XL mass spectrometer (Thermo Electron Corp., Bremen, Germany) equipped with an online nano-electrospray ion source (Michrom Bioresources, Auburn, USA).

A 180 µm×2.4 cm SCX column (Waters Corporation, Milford, USA), which was packed with a 5 µm Poly Sulfoethyl Aspartamide (PolyLC, Columbia, MD, USA) was used for the first dimension. To recover hydrophobic peptides still retained on the SCX column after a conventional salt step gradient, a RP step gradient from 5% to 50% acetonitrile (ACN) was applied to the SCX column. A 15 µl plug was injected each time to form the step gradients. At last, 1 M Ammonium formate (NH4FA) was used to clean the SCX colum once. The plugs were loaded onto the SCX column with a loading buffer at a 15 µl/min flow rate for 6 min. A 15 µl peptide sample was loaded onto the SCX column before the gradient plugs were injected. The eluted peptides were captured by a trap column (Waters) while salts were diverted to waste. The trap column (2 cm x 180 µm) was packed with a 5 µm Symmetry C18 material (Waters). The RP analytical column (15 cm x 100 µm) was packed with a 1.7 µm Bridged Ethyl Hybrid (BEH) C18 material (Waters), and was used for the second dimension separation.

The peptides on the RP analytical column were eluted with a three-step linear gradient. Starting from 5% B to 40% B in 40 min (A: water with 0.1% formic acid; B: ACN with 0.1% formic acid), increased to 80% B in 3 min, and then to 5% B in 2 min. The column was re-equilibrated at initial conditions for 15 min. The column flow rate was maintained at 500 nl/min and column temperature was maintained at 35°C. The electrospray voltage of 1.9 kV versus the inlet of the mass spectrometer was used.

LTQ Orbitrap XL mass spectrometer was operated in the data-dependent mode to switch automatically between MS and MS/MS acquisition. Survey full-scan MS spectra with two microscans (m/z 300–1800) were acquired in the Obitrap with a mass resolution of 60,000 at m/z 400, followed by ten sequential LTQ-MS/MS scans. Dynamic exclusion was used with two repeat counts, 10 s repeat duration, and 90 s exclusion duration. For MS/MS, precursor ions were activated using 35% normalized collision energy at the default activation q of 0.25.

The 2D-LC-MS/MS experiment was repeat three times for cancer sample and corresponding adjacent noncancerous sample, respectively.

### Peptide sequencing and data analysis

All MS/MS spectrums were identified by using SEQUEST [v.28 (revision 12), Thermo Electron Corp.] against the human UniProtKB/Swiss-Prot database (Release 2011_12_14, with 20249 entries), as previously described [12]. To reduce false positive identification results, a decoy database containing the reverse sequences was appended to the database. The searching parameters were set up as follows: full trypsin cleavage with two missed cleavage was considered, the variable modification was oxidation of methionine, the peptide mass tolerance was 20 ppm, and the fragment ion tolerance was 1 Da. Trans Proteomic Pipeline software (revision 4.0)(Institute of Systems Biology, Seattle, WA) was then utilized to identify proteins based upon corresponding peptide sequences with ≥95% confidence. The peptides results were filtered by Peptide Prophet with a p-value over 0.90 and a Protein Prophet probability of 0.95 was used for the protein identification results. Employing the APEX tool to quantified the protein abundances, the abundances estimated by normalizing for the measured total protein concentration. The false positive rate of less than 1% was set for all peptide identifications.

### Bioinformatics analysis

The original data were derived from analysis using APEX software. Differentially expressed proteins were screened using the 2-sample t-test (P<0.05) and fold change (>1.5 or <0.667) method. All expression values of the differentially expressed proteins were first converted to a log form and then input as hierarchical clustering algorithms, where the Euclidean distance was used for distance and average for linkage for GO analysis. Differentially expressed genes were mapped to the appropriate GO database to calculate the number of genes at each node, using EASE software. The differentially expressed genes were classified according to bp (biologic process), cc (cellular component), and mf (molecular function) independently. In protein network analysis, interactions between genes in the range of the genomes analyzed were analyzed by downloading the pathway data in KEGG, MIPS, PubMed, MINT, Human Protein Reference Database (HPRD), BioGRID, Database of Interacting Proteins (DIP), and Reactome, using the BIND software package. Interrelationships between genes that had been reported in the literature were analyzed by co-citation calculation. The established gene network was able to directly reflect the interrelationships between genes at an overall level as well as the stability of the gene regulatory network.

### Cell line and culture

The human laryngeal carcinoma cell line Hep-2 was obtained from the cell bank of the Shanghai Institute of Cell Biology (Shanghai, China). The cells were maintained in RPMI 1640 upplemented with 10% FBS, 100 U/ml penicillin, 100 µg/ml streptomycin sulphate, and 1 mM sodium pyruvate at 37°C in 5% CO_2_.

### siRNAs preparation and transfection

The siRNAs were chemically synthesised by Shanghai GenePharma Co., Ltd.. The siRNA sequences for PFN1 were previously described [19]: siRNA-PFN1: 5′- AGA AGG UGU CCA CGG UGG UUU -3′ (forward) and 5′- ACC ACC GUG GAC ACC UUC UUU -3′ (reverse). The negative control siRNAs were designed as follows: 5′-UAG CGA CUA AAC ACA UCA AUU-3′ (forward) and 5′-UUG AUG UGU UUA GUC GCU AUU-3′ (reverse). According to the manufacturer's specifications, the transfections of siRNA were carried out with Lipo2000 (Invitrogen) in 6-well plates. Until reached 50–70% confluence, the Hep-2 cells were transfected with 20 nM of siRNA for 6–12 h, and then replaced with the regular growth media. And cells were cultured for another 24–72 h before performing the experiments.

### Semiquantitative RT-PCR

The total RNA was isolated from frozen tissues, and cells were extracted using TRIzol reagent (Takara). Two microgram of total RNA was used for cDNA synthesis using the RevertAidtm First Strand cDNA Synthesis Kit #1622 (Fermentas) according to the manufacturer's instructions. The primer sequences and the expected sizes of PCR products were as follows: PFN1, 5′-ATC GAC AAC CTC ATG GCG GAC G-3′(forward) and 5′-TTG CCA ACC AGG ACA CCC ACC T-3′(reverse) (140 bp); NCL, 5′-GAA AGC GTT GGA ACT CAC-3′(forward) and 5′-AAG TGT TCT CGC ATC TCG-3′(reverse) (103 bp); CNDP2, 5′-AAC TCA GGC CCT CCC TCT GTT GT-3′(forward) and 5′-GCT CCA GGA AGT GAC TGC GGC-3′(reverse) (146 bp); OGN, 5′- GTT GAC ATT GAT GCT GTA CCA CCC-3′(forward) and 5′-GCT TGG GAG GAA GAA CTG GA-3′(reverse) (241 bp). GAPDH, 5′-CAA GGT CAT CCA TGA CAA CTT TG-3′ (forward) and 5′-GTC CAC CAC CCT GTT GCT GTA G-3′(reverse) (496 bp). The PCR conditions used for the amplification were as follows: 94°C for 5 min, then 30 cycles of 94°C for 20 s, 55–60°C for 20 s, and 72°C for 30 s, followed by 72°C for 10 min. The RT-PCR products were analysed on a 1% agarose gel and visualised with ethidium bromide staining. The GAPDH gene was used as a positive control to assess the cDNA quality.

### Cell proliferation assay

Cells (1×10^4^/ml) were plated in 96-well plates. At 24, 48, and 72 h post-transfection with PFN1 siRNA, the cell viability was determined by cell counting kit-8 (CCK-8) assay (Dojindo) according to the manufacture's protocol.

### Transwell assay

Transwell assay was performed using polycarbonate transwell filters (Corning, 8 µm) as previously described [20]. Briefly, at 12 h posttransfection, a sample of 0.8×10^5^ cells were suspended in medium containing 1% FBS and added to the upper chamber. And the bottom chambers were filled with culture medium containing 20% FBS. After incubation for 24 h, the cells on the upper surface of the well were removed, and the cells on the lower surface were fixed in cold methanol and stained with 0.4% crystal violet (Sigma). For each experiment, the number of transmigrated cells in five random fields on the underside of the filter was counted and photographed, and three independent filters were analysed.

### Western blotting

Whole-cell lysates were prepared from human tissue specimens and treated cells. For Western blotting analysis, equal amounts of proteins were separated using SDS-PAGE and transferred to a nitrocellulose membrane and then incubated with monoclonal antibody anti-PFN1 (Epitomics), monoclonal antibody anti-NCL (Santa Cruz), polyclonal antibody anti-CNDP2 (Proteintech), polyclonal antibody anti-OGN (Abgent), or monoclonal antibody anti-GAPDH (Bioworld) at 4°C overnight. The immunocomplexes were visualised using a horseradish peroxidase-conjugated antibody followed by a chemoluminescence reagent (Millipore) and detected on photographic film.

### Statistical analysis

The data was expressed as the mean ± SD. All calculations were performed with SPSS version 11.7. The statistical analyses were performed with Student's t-test and analysis of variance. Multiple groups comparison in other assays was performed by one-way ANOVA.

All P values were two tailed, and <0.05 was considered statistically significant.

## Results

### Screening for differentially expressed proteins

**Figure 1:**
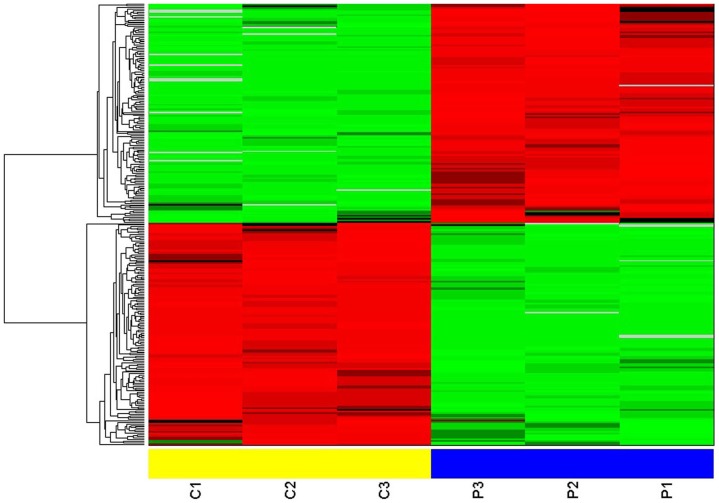
Hierarchical cluster analysis of the proteins expressed with statistically significant differences (P<0.05, and fold change >1.5 or <0.667) in cancer tissue and paracancerous normal tissue from patients with laryngeal carcinoma. Three independent experiments were performed in cancer tissue (C1, C2, C3) and paracancerous normal tissue (P1, P2, P3).

Using APEX software, the original data were analyzed. Three independent experiments were performed in the laryngeal carcinoma (C) and the corresponding adjacent noncancerous (P) samples pools, respectively. According to the stringent criteria of having >1 unique peptide per protein present and a false discovery rate of ≤5%, 1,738 proteins were identified from the two sample pools. Following the statistical Student's 2-sample t-test analysis and the Fold change (C/P) methods, 141 proteins were significantly up-regulated using the criteria of P<0.05 and fold change >1.5, and 140 proteins were significantly down-regulated by P<0.05 and fold change <0.667 (Table 2). The expression values of the expressed proteins with significant difference were first converted to a log form and then input as hierarchical cluster algorithms. The results are shown in Figure 1.

**Table 2:** Differentially expressed proteins screened out compared the laryngeal carcinoma tissues (C) with the corresponding adjacent noncancerous tissues (P).

Uniprot ID	Identified Proteins	Gene name	Fold change (C/P)	T test
P29508	Serpin B3	SERPINB3	15.30	0.00677
P53634	Dipeptidyl-peptidase 1	CTSC	13.70	0.00474
P02792	Ferritin light chain	FTL	9.91	0.01259
P04899	Guanine nucleotide-binding protein G(i), alpha-2 subunit	GNAI2	9.77	0.00089
Q15181	Inorganic pyrophosphatase	PPA1	8.51	0.01315
P19971	Thymidine phosphorylase	TYMP	8.06	0.00101
P40227	T-complex protein 1 subunit zeta	CCT6A	7.86	0.00698
P59998	Actin-related protein 2/3 complex subunit 4	ARPC4	7.64	0.00176
P50552	Vasodilator-stimulated phosphoprotein	VASP	7.25	0.01336
P13797	Plastin-3	PLS3	6.52	0.00256
P09467	Fructose-1,6-bisphosphatase 1	FBP1	6.37	0.04326
O00299	Chloride intracellular channel protein 1	CLIC1	5.91	0.00516
P52895	Aldo-keto reductase family 1 member C2	AKR1C2	5.82	0.0004
O15533	Tapasin	TAPBP	5.81	0.01439
Q16630	Cleavage and polyadenylation specificity factor subunit 6	CPSF6	5.76	0.04131
P54578	Ubiquitin carboxyl-terminal hydrolase 14	USP14	5.47	0.01414
P07737	Profilin-1	PFN1	5.20	0.00692
P37837	Transaldolase	TALDO1	5.12	0.0124
P31939	Bifunctional purine biosynthesis protein PURH	ATIC	5.05	0.01149
P35637	RNA-binding protein FUS	FUS	5.02	0.01108
P47929	Galectin-7	LGALS7	4.92	0.00664
O00764	Pyridoxal kinase	PDXK	4.91	0.0044
P23141	Liver carboxylesterase 1	CES1	4.86	0.01626
A6NIZ1	Ras-related protein Rap-1b	RAP1B	4.78	0.00595
P42224	Signal transducer and activator of transcription 1-alpha/beta	STAT1	4.60	0.00168
P55209	Nucleosome assembly protein 1-like 1	NAP1L1	4.41	0.02318
O14979	Heterogeneous nuclear ribonucleoprotein D-like	HNRPDL	4.34	0.03791
P11413	Glucose-6-phosphate 1-dehydrogenase	G6PD	4.30	0.01466
P58107	Epiplakin	EPPK1	4.30	0.0157
Q96AB3	Isochorismatase domain-containing protein 2, mitochondrial	ISOC2	4.26	0.01074
P28838	Cytosol aminopeptidase	LAP3	4.25	0.01448
O75569	Interferon-inducible double stranded RNA-dependent protein kinase activator A	PRKRA	4.21	0.04359
Q12874	Splicing factor 3A subunit 3	SF3A3	4.18	0.01359
Q96HE7	ERO1-like protein alpha	ERO1L	4.09	0.02022
O60664	Mannose-6-phosphate receptor-binding protein 1	M6PRBP1	3.91	0.00369
P99999	Cytochrome c	CYCS	3.91	0.00451
P19338	Nucleolin	NCL	3.90	0.02815
O75874	Isocitrate dehydrogenase [NADP] cytoplasmic	IDH1	3.81	0.01901
P23246	Splicing factor, proline- and glutamine-rich	SFPQ	3.67	0.00085
Q01518	Adenylyl cyclase-associated protein 1	CAP1	3.61	0.01283
Q07666	KH domain-containing, RNA-binding, signal transduction-associated protein 1	KHDRBS1	3.55	0.0109
Q96KP4	Cytosolic non-specific dipeptidase	CNDP2	3.50	0.01266
P30043	Flavin reductase	BLVRB	3.46	0.02879
P05164	Myeloperoxidase	MPO	3.44	0.00316
P36871	Phosphoglucomutase-1	PGM1	3.44	0.03173
P50995	Annexin A11	ANXA11	3.43	0.03113
P02786	Transferrin receptor protein 1	TFRC	3.40	0.04239
Q16658	Fascin	FSCN1	3.36	0.01824
P63104	14-3-3 protein zeta/delta	YWHAZ	3.35	0.01256
P00491	Purine nucleoside phosphorylase	NP	3.32	0.00487
Q9UNM6	26S proteasome non-ATPase regulatory subunit 13	PSMD13	3.28	0.04739
P68104	Putative elongation factor 1-alpha-like 3	EEF1AL3	3.28	0.00105
Q13347	Eukaryotic translation initiation factor 3 subunit I	EIF3I	3.20	0.01014
Q96FQ6	Protein S100-A16	S100A16	3.17	0.01151
P52565	Rho GDP-dissociation inhibitor 1	ARHGDIA	3.15	0.00299
Q99715	Collagen alpha-1(XII) chain	COL12A1	3.12	0.02148
P29401	Transketolase	TKT	3.09	0.00809
P53582	Methionine aminopeptidase 1	METAP1	3.08	0.00304
P52209	6-phosphogluconate dehydrogenase, decarboxylating	PGD	2.96	0.03408
P17096	High mobility group protein HMG-I/HMG-Y	HMGA1	2.95	0.02965
P13693	Translationally-controlled tumor protein	TPT1	2.88	0.02137
Q01469	Fatty acid-binding protein, epidermal	FABP5	2.86	0.03051
Q14974	Importin subunit beta-1	KPNB1	2.84	0.01796
P31949	Protein S100-A11	S100A11	2.82	0.01872
P02144	Myoglobin	MB	2.81	0.01049
Q16543	Hsp90 co-chaperone Cdc37	CDC37	2.77	0.01228
P50914	60S ribosomal protein L14	RPL14	2.75	0.00198
Q9Y490	Talin-1	TLN1	2.67	0.0068
P06733	Alpha-enolase	ENO1	2.64	0.01238
P24821	Tenascin	TNC	2.63	0.01489
P37802	Transgelin-2	TAGLN2	2.57	0.03438
P61160	Actin-related protein 2	ACTR2	2.57	0.00239
P30838	Aldehyde dehydrogenase, dimeric NADP-preferring	ALDH3A1	2.56	0.03337
P13010	ATP-dependent DNA helicase 2 subunit 2	XRCC5	2.55	0.00755
P30085	UMP-CMP kinase	CMPK1	2.55	0.0115
Q9UN86	Ras GTPase-activating protein-binding protein 2	G3BP2	2.55	0.01204
P18206	Vinculin	VCL	2.53	0.02193
Q92616	Translational activator GCN1	GCN1L1	2.53	0.02057
P05198	Eukaryotic translation initiation factor 2 subunit 1	EIF2S1	2.50	0.03971
O00303	Eukaryotic translation initiation factor 3 subunit F	EIF3F	2.49	0.01838
P09110	3-ketoacyl-CoA thiolase, peroxisomal	ACAA1	2.49	0.00319
P09651	Heterogeneous nuclear ribonucleoprotein A1	HNRNPA1	2.49	0.04643
P63241	Eukaryotic translation initiation factor 5A-1	EIF5A	2.48	0.00699
P16401	Histone H1.5	HIST1H1B	2.47	0.02978
P60842	Eukaryotic initiation factor 4A-I	EIF4A1	2.47	0.02891
P04406	Glyceraldehyde-3-phosphate dehydrogenase	GAPDH	2.43	0.01183
P07195	L-lactate dehydrogenase B chain	LDHB	2.41	0.0067
Q12931	Heat shock protein 75 kDa, mitochondrial	TRAP1	2.40	0.00724
Q99832	T-complex protein 1 subunit eta	CCT7	2.40	0.00158
P18669	Phosphoglycerate mutase 1	PGAM1	2.39	0.02256
Q07960	Rho GTPase-activating protein 1	ARHGAP1	2.37	0.00799
P60174	Triosephosphate isomerase	TPI1	2.36	0.00203
P13639	Elongation factor 2	EEF2	2.35	0.00292
P26641	Elongation factor 1-gamma	EEF1G	2.34	0.00212
Q86VP6	Cullin-associated NEDD8-dissociated protein 1	CAND1	2.29	0.00207
P38606	V-type proton ATPase catalytic subunit A	ATP6V1A	2.26	0.03249
Q15691	Microtubule-associated protein RP/EB family member 1	MAPRE1	2.23	0.03292
P62244	40S ribosomal protein S15a	RPS15A	2.19	0.01589
P69905	Hemoglobin subunit alpha	HBA1	2.19	0.00953
Q07021	Complement component 1 Q subcomponent-binding protein, mitochondrial	C1QBP	2.17	0.03131
P02751	Fibronectin	FN1	2.16	0.01678
P09211	Glutathione S-transferase P	GSTP1	2.15	0.02025
P23381	Tryptophanyl-tRNA synthetase, cytoplasmic	WARS	2.13	0.00901
P26599	Polypyrimidine tract-binding protein 1	PTBP1	2.13	0.03262
P29692	Elongation factor 1-delta	EEF1D	2.13	0.0437
P14618	Pyruvate kinase isozymes M1/M2	PKM2	2.13	0.00143
P11940	Polyadenylate-binding protein 1	PABPC1	2.12	0.02495
P13667	Protein disulfide-isomerase A4	PDIA4	2.11	0.01605
P13796	Plastin-2	LCP1	2.11	0.00619
P09960	Leukotriene A-4 hydrolase	LTA4H	2.10	0.01662
Q15149	Plectin-1	PLEC1	2.07	0.00424
Q13011	Delta(3,5)-Delta(2,4)-dienoyl-CoA isomerase, mitochondrial	ECH1	2.06	0.01475
P04632	Calpain small subunit 1	CAPNS1	2.06	0.03241
P00918	Carbonic anhydrase 2	CA2	2.05	0.0467
P40616	ADP-ribosylation factor-like protein 1	ARL1	2.02	0.01116
P40939	Trifunctional enzyme subunit alpha, mitochondrial	HADHA	2.01	0.00595
P23528	Cofilin-1	CFL1	2.01	0.02903
Q8NBS9	Thioredoxin domain-containing protein 5	TXNDC5	1.93	0.00024
P52566	Rho GDP-dissociation inhibitor 2	ARHGDIB	1.92	0.04385
Q14103	Heterogeneous nuclear ribonucleoprotein D0	HNRNPD	1.92	0.03422
Q08211	ATP-dependent RNA helicase A	DHX9	1.91	0.01272
Q8TE68	Epidermal growth factor receptor kinase substrate 8-like protein 1	EPS8L1	1.91	0.02329
O15372	Eukaryotic translation initiation factor 3 subunit H	EIF3H	1.85	0.01438
P07858	Cathepsin B	CTSB	1.84	0.00487
P17655	Calpain-2 catalytic subunit	CAPN2	1.84	0.03102
P10809	60 kDa heat shock protein, mitochondrial	HSPD1	1.82	0.02513
P51970	NADH dehydrogenase [ubiquinone] 1 alpha subcomplex subunit 8	NDUFA8	1.82	0.04023
P14625	Endoplasmin	HSP90B1	1.82	0.0194
P30153	Serine/threonine-protein phosphatase 2A 65 kDa regulatory subunit A alpha isoform	PPP2R1A	1.81	0.0417
O75955	Flotillin-1	FLOT1	1.78	0.00694
Q02878	60S ribosomal protein L6	RPL6	1.78	0.01691
O75083	WD repeat-containing protein 1	WDR1	1.75	0.01675
P12268	Inosine-5′-monophosphate dehydrogenase 2	IMPDH2	1.75	0.0418
P24158	Myeloblastin	PRTN3	1.74	0.01096
Q7KZF4	Staphylococcal nuclease domain-containing protein 1	SND1	1.73	0.00169
P00558	Phosphoglycerate kinase 1	PGK1	1.72	0.00095
P60660	Myosin light polypeptide 6	MYL6	1.70	0.02066
Q13200	26S proteasome non-ATPase regulatory subunit 2	PSMD2	1.69	0.04337
P61019	Ras-related protein Rab-2A	RAB2A	1.65	0.0369
Q92597	Protein NDRG1	NDRG1	1.61	0.00689
P02768	Serum albumin	ALB	1.57	0.00957
O00231	26S proteasome non-ATPase regulatory subunit 11	PSMD11	1.56	0.02677
Q9P2E9	Ribosome-binding protein 1	RRBP1	0.66	0.02565
P98160	Basement membrane-specific heparan sulfate proteoglycan core protein	HSPG2	0.65	0.02063
P06576	ATP synthase subunit beta, mitochondrial	ATP5B	0.65	0.04632
Q9ULV4	Coronin-1C	CORO1C	0.58	0.04756
P29966	Myristoylated alanine-rich C-kinase substrate	MARCKS	0.58	0.01746
P62861	40S ribosomal protein S30	FAU	0.57	0.00947
P06396	Gelsolin	GSN	0.56	0.04161
P02652	Apolipoprotein A-II	APOA2	0.56	0.04
P02763	Alpha-1-acid glycoprotein 1	ORM1	0.55	0.00937
P31146	Coronin-1A	CORO1A	0.55	0.034
P11047	Laminin subunit gamma-1	LAMC1	0.54	0.02215
P20700	Lamin-B1	LMNB1	0.53	0.03269
P10412	Histone H1.2	HIST1H1C	0.53	0.01299
P14866	Heterogeneous nuclear ribonucleoprotein L	HNRNPL	0.53	0.00906
P09622	Dihydrolipoyl dehydrogenase, mitochondrial	DLD	0.53	0.00322
P14923	Junction plakoglobin	JUP	0.52	0.01465
O95994	Anterior gradient protein 2 homolog	AGR2	0.52	0.04294
P49748	Very long-chain specific acyl-CoA dehydrogenase, mitochondrial	ACADVL	0.52	0.03597
P08603	Complement factor H	CFH	0.52	0.01851
Q16891	Mitochondrial inner membrane protein	IMMT	0.51	0.03273
P05091	Aldehyde dehydrogenase, mitochondrial	ALDH2	0.51	0.02974
P00505	Aspartate aminotransferase, mitochondrial	GOT2	0.50	0.00699
Q13813	Spectrin alpha chain, brain	SPTAN1	0.50	0.00471
P11216	Glycogen phosphorylase, brain form	PYGB	0.50	0.02901
Q6YN16	Hydroxysteroid dehydrogenase-like protein 2	HSDL2	0.50	0.00185
P10155	60 kDa SS-A/Ro ribonucleoprotein	TROVE2	0.50	0.03947
P30084	Enoyl-CoA hydratase, mitochondrial	ECHS1	0.50	0.0033
P19823	Inter-alpha-trypsin inhibitor heavy chain H2	ITIH2	0.49	0.02225
P30048	Thioredoxin-dependent peroxide reductase, mitochondrial	PRDX3	0.49	0.01532
P08727	Keratin, type I cytoskeletal 19	KRT19	0.49	0.01893
Q9UHG3	Prenylcysteine oxidase 1	PCYOX1	0.49	0.02023
P27635	60S ribosomal protein L10	RPL10	0.49	0.02615
P27824	Calnexin	CANX	0.49	0.04302
Q15582	Transforming growth factor-beta-induced protein ig-h3	TGFBI	0.49	0.00143
P01024	Complement C3	C3	0.49	0.00256
P00488	Coagulation factor XIII A chain	F13A1	0.49	0.01016
P02747	Complement C1q subcomponent subunit C	C1QC	0.48	0.04694
Q01082	Spectrin beta chain, brain 1	SPTBN1	0.48	0.01722
P39656	Dolichyl-diphosphooligosaccharide–protein glycosyltransferase 48 kDa subunit	DDOST	0.48	0.04726
P04080	Cystatin-B	CSTB	0.47	0.04055
P01023	Alpha-2-macroglobulin	A2M	0.47	0.02189
Q9NSE4	Isoleucyl-tRNA synthetase, mitochondrial	IARS2	0.46	0.04337
P36269	Gamma-glutamyltransferase 5	GGT5	0.46	0.00259
P21810	Biglycan	BGN	0.46	0.00989
P31040	Succinate dehydrogenase flavoprotein subunit, mitochondrial	SDHA	0.45	0.00591
P02788	Lactotransferrin	LTF	0.45	0.02393
P62158	Calmodulin	CALM1	0.44	0.01293
P01857	Ig gamma-1 chain C region	IGHG1	0.43	0.00036
P22695	Cytochrome b-c1 complex subunit 2, mitochondrial	UQCRC2	0.43	0.00169
P46781	40S ribosomal protein S9	RPS9	0.43	0.04449
Q02218	2-oxoglutarate dehydrogenase E1 component, mitochondrial	OGDH	0.43	0.01132
P24539	ATP synthase subunit b, mitochondrial	ATP5F1	0.42	0.01379
P17931	Galectin-3	LGALS3	0.42	0.00253
P01009	Alpha-1-antitrypsin	SERPINA1	0.41	0.00246
P00738	Haptoglobin	HP	0.41	0.0076
P62280	40S ribosomal protein S11	RPS11	0.41	0.04435
P43304	Glycerol-3-phosphate dehydrogenase, mitochondrial	GPD2	0.41	0.02182
O60716	Catenin delta-1	CTNND1	0.41	0.01993
Q96IU4	Abhydrolase domain-containing protein 14B	ABHD14B	0.40	0.04326
Q14152	Eukaryotic translation initiation factor 3 subunit A	EIF3A	0.40	0.04596
P51888	Prolargin	PRELP	0.40	0.0218
P02511	Alpha-crystallin B chain	CRYAB	0.40	0.02841
Q8NCW5	Apolipoprotein A-I-binding protein	APOA1BP	0.39	0.014
P84098	60S ribosomal protein L19	RPL19	0.39	0.01034
O75306	NADH dehydrogenase iron-sulfur protein 2, mitochondrial	NDUFS2	0.39	0.0012
P07099	Epoxide hydrolase 1	EPHX1	0.39	0.01386
P49755	Transmembrane emp24 domain-containing protein 10	TMED10	0.39	0.03179
P60709	Actin, cytoplasmic 2	ACTG1	0.39	0.00425
P00450	Ceruloplasmin	CP	0.38	0.01124
P21796	Voltage-dependent anion-selective channel protein 1	VDAC1	0.38	0.02369
P02545	Lamin-A/C	LMNA	0.38	0.00148
P39059	Collagen alpha-1(XV) chain	COL15A1	0.38	0.02395
P63167	Dynein light chain 1, cytoplasmic	DYNLL1	0.38	0.00646
Q14134	Tripartite motif-containing protein 29	TRIM29	0.38	0.00844
P51571	Translocon-associated protein subunit delta	SSR4	0.37	0.02087
Q9UN36	Protein NDRG2	NDRG2	0.37	0.02335
P01834	Ig kappa chain C region	IGKC	0.37	0.03372
Q02790	FK506-binding protein 4	FKBP4	0.37	0.01019
P04217	Alpha-1B-glycoprotein	A1BG	0.36	0.02191
Q9BS26	Thioredoxin domain-containing protein 4	TXNDC4	0.36	0.02078
P30040	Endoplasmic reticulum protein ERp29	ERP29	0.35	0.0485
P12532	Creatine kinase, ubiquitous mitochondrial	CKMT1A	0.35	0.02472
Q08380	Galectin-3-binding protein	LGALS3BP	0.35	0.00749
P30049	ATP synthase subunit delta, mitochondrial	ATP5D	0.33	0.01374
P63244	Guanine nucleotide-binding protein subunit beta-2-like 1	GNB2L1	0.33	0.002
P35232	Prohibitin	PHB	0.33	0.01226
Q01081	Splicing factor U2AF 35 kDa subunit	U2AF1	0.31	0.01422
Q9UQ80	Proliferation-associated protein 2G4	PA2G4	0.31	0.01201
P02730	Band 3 anion transport protein	SLC4A1	0.30	0.01198
P31930	Cytochrome b-c1 complex subunit 1, mitochondrial	UQCRC1	0.29	0.00459
O75367	Core histone macro-H2A.1	H2AFY	0.29	0.00555
P11177	Pyruvate dehydrogenase E1 component subunit beta, mitochondrial	PDHB	0.29	0.04049
P24752	Acetyl-CoA acetyltransferase, mitochondrial	ACAT1	0.28	0.01184
P08294	Extracellular superoxide dismutase [Cu-Zn]	SOD3	0.27	0.04222
Q05707	Collagen alpha-1(XIV) chain	COL14A1	0.27	0.00088
P62826	GTP-binding nuclear protein Ran	RAN	0.27	0.03551
Q99623	Prohibitin-2	PHB2	0.27	0.00683
P04844	Dolichyl-diphosphooligosaccharide—protein glycosyltransferase subunit 2	RPN2	0.26	0.02265
Q12907	Vesicular integral-membrane protein VIP36	LMAN2	0.26	0.02661
P04181	Ornithine aminotransferase, mitochondrial	OAT	0.26	0.01802
P62750	60S ribosomal protein L23a	RPL23A	0.25	0.0077
P06732	Creatine kinase M-type	CKM	0.25	0.02901
P14927	Cytochrome b-c1 complex subunit 7	UQCRB	0.25	0.04749
P51884	Lumican	LUM	0.25	0.0166
Q9UH99	Protein unc-84 homolog B	UNC84B	0.24	0.01415
P00403	Cytochrome c oxidase subunit 2	MT-CO2	0.24	0.00441
P02675	Fibrinogen beta chain	FGB	0.24	0.01004
P49257	Protein ERGIC-53	LMAN1	0.23	0.00243
Q03252	Lamin-B2	LMNB2	0.23	0.00382
P58546	Myotrophin	MTPN	0.23	0.03507
P32969	60S ribosomal protein L9	RPL9	0.22	0.03767
O95299	NADH dehydrogenase [ubiquinone] 1 alpha subcomplex subunit 10, mitochondrial	NDUFA10	0.21	0.02844
Q16795	NADH dehydrogenase [ubiquinone] 1 alpha subcomplex subunit 9, mitochondrial	NDUFA9	0.21	0.01163
P04843	Dolichyl-diphosphooligosaccharide–protein glycosyltransferase subunit 1	RPN1	0.20	0.00436
Q8TDL5	Long palate, lung and nasal epithelium carcinoma-associated protein 1	LPLUNC1	0.20	0.02273
P20774	Mimecan	OGN	0.20	0.03045
P02679	Fibrinogen gamma chain	FGG	0.19	0.00129
P48047	ATP synthase subunit O, mitochondrial	ATP5O	0.19	0.01449
P62841	40S ribosomal protein S15	RPS15	0.19	0.02324
Q92817	Envoplakin	EVPL	0.19	0.00422
P09493	Tropomyosin alpha-1 chain	TPM1	0.19	0.01514
P19652	Alpha-1-acid glycoprotein 2	ORM2	0.18	0.00543
Q9UIJ7	GTP:AMP phosphotransferase mitochondrial	AK3	0.18	0.01683
P00367	Glutamate dehydrogenase 1, mitochondrial	GLUD1	0.18	0.01563
P10916	Myosin regulatory light chain 2, ventricular/cardiac muscle isoform	MYL2	0.18	0.00277
P00387	NADH-cytochrome b5 reductase 3	CYB5R3	0.17	0.00535
Q9UI09	NADH dehydrogenase 1 alpha subcomplex subunit 12	NDUFA12	0.17	0.00843
P32322	Pyrroline-5-carboxylate reductase 1, mitochondrial	PYCR1	0.17	0.00997
P45880	Voltage-dependent anion-selective channel protein 2	VDAC2	0.17	0.00812
Q9BSJ8	Extended synaptotagmin-1	FAM62A	0.17	0.02282
P12883	Myosin-7	MYH7	0.15	0.04314
P07585	Decorin	DCN	0.15	0.00903
P45378	Troponin T, fast skeletal muscle	TNNT3	0.14	0.03654
Q07954	Prolow-density lipoprotein receptor-related protein 1	LRP1	0.13	0.01165
P61626	Lysozyme C	LYZ	0.13	0.00319
P20618	Proteasome subunit beta type-1	PSMB1	0.11	0.03038
Q96A32	Myosin regulatory light chain 2, skeletal muscle isoform	MYLPF	0.09	0.00292
P01876	Ig alpha-1 chain C region	IGHA1	0.07	0.00055
Q9BXN1	Asporin	ASPN	0.07	0.00292
P35749	Myosin-11	MYH11	0.06	0.03254

### GO analysis of the proteins with significant difference in expression

**Figure 2:**
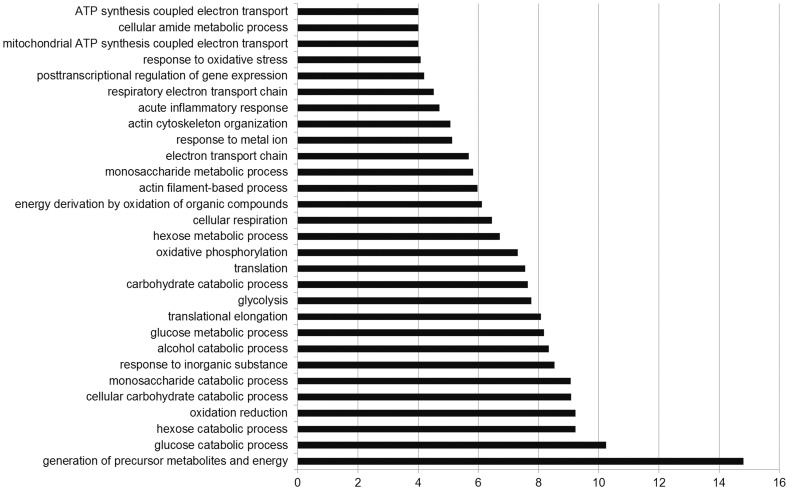
Gene ontology analysis of differentially expressed proteins classified according to biologic process.

To get more insight on the biological significance of the differentially expressed proteins in human laryngeal carcinoma, GO analysis was conducted on 281 differentially expressed proteins (Figure 2). According to biologic process analysis, it showed that each group was enriched with the proteins of different functions, suggesting that the differentially expressed proteins may play a distinctive role in human laryngeal carcinogenesis by these signaling pathways.

### Analysis of the differential protein network

**Figure 3:**
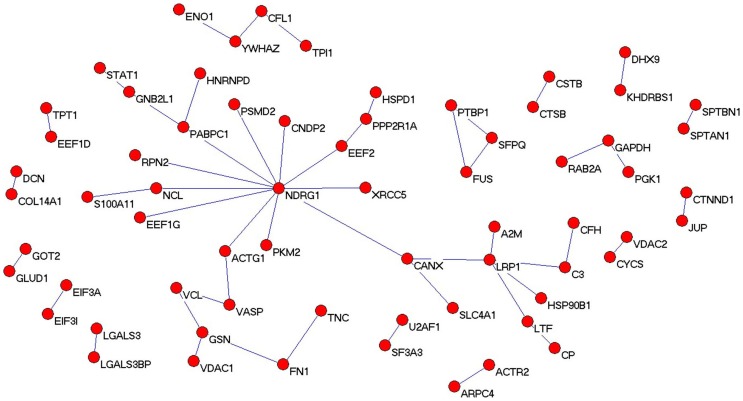
Protein network analysis. The protein–protein interaction network of differential proteins is shown.

To identify the potential interrelationships between proteins expressed with significant difference, a protein–protein interaction network was built up with Pajek software. Consistently, the differential protein network was established by integrating three different types of interaction: 1) protein–protein interactions obtained in well established high-throughput experiments such as yeast 2-hybrid experiments; 2) gene interactions reported in the literature; and 3) protein interaction, gene regulation, and protein decoration. The results are shown in Figure 3. These proteins may have important roles in laryngeal carcinoma oncogenesis and progression, and their presence in this network diagram confirms the relevance of the differentially expressed proteins data set and their association to laryngeal carcinoma, in some way.

### Validation of differentially expressed proteins indentified by proteomics

**Figure 4:**
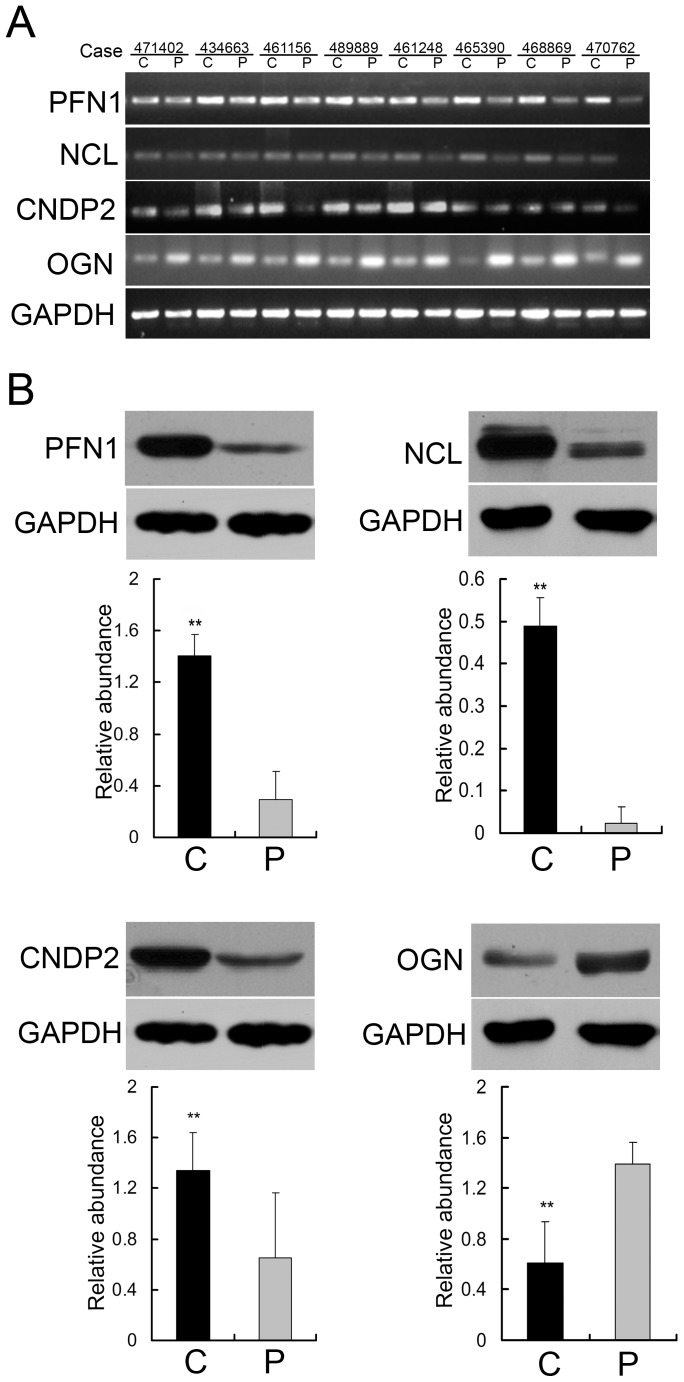
Validation of differentially expressed proteins in laryngeal carcinoma tissue and the adjacent normal tissue by semiquantitative RT-PCR and Western blotting. (A) The representative image of mRNA levels of PFN1, NCL, CNDP2 and OGN between laryngeal carcinoma tissue and their corresponding normal tissue in 8 cases of tissues measured by semiquantitative RT-PCR. (B) The representative result of Western blotting show the expressions of PFN1, NCL, CNDP2 and OGN in the laryngeal carcinoma tissue and the adjacent normal tissue, respectively. Histograms are representative the relative abundance of proteins mean from 24 cases of tissues. (**P<0.01 by One-way ANOVA).

Next, semiquantitative RT-PCR was performed to detect the mRNA levels of PFN1, NCL, CNDP2 and OGN in 8 cases of paired laryngeal carcinoma tissues. As shown in Figure 4A, in most cases, PFN1, NCL and CNDP2 exerted an increased mRNA expression, while OGN displayed a decreased level in the carcinoma tissues compared with the adjacent normal tissue. The protein expression level of the four selected molecules was further investigated with 24 paired cases of laryngeal carcinoma and non-cancer tissue sections using Western blotting. And the results revealed that the expression of PFN1, NCL, and CNDP2 was elevated and that of OGN was reduced in laryngeal carcinoma tissue compared with the adjacent normal tissue (Figure 4B). Thus, these results validate the differentially expressed proteins indentified by the proteomics.

### PFN1 silencing inhibits the proliferation and metastasis of the human laryngeal carcinoma Hep-2 cells

**Figure 5:**
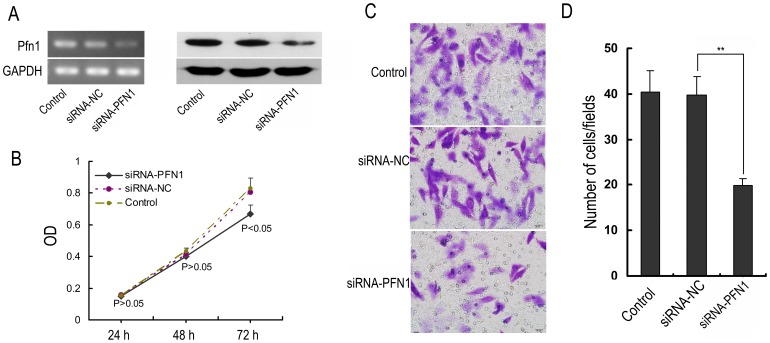
Effects of PFN1 silencing on the proliferation and metastasis of Hep-2 cells. (A) The mRNA (24 h) and protein (48 h) expression of PFN1 after specific siRNA transfection in Hep-2 cells. The levels of mRNA and protein were determined by semiquantitative RT-PCR and Western blotting, respectively. (B) The cell viability of Hep-2 cells harvested 24, 48, and 72 h post-transfection after treatment with siPFN1. The optical density (OD) represents the proliferative characters of the treated cells. (C) The directed migratory capacities of Hep-2 cells after the siPFN1 transfected for 24 h were evaluated using a Transwell migration study. Images of cells on the undersurface of a filter are shown. Bar, 20 µm. (D) The number of cells per field in control and treated cells is shown. Values are the mean ± SD from three independent experiments. **P<0.01.

To know whether down-regulation of PFN1 is involved in laryngeal carcinoma carcinogenesis, Hep-2 cells were transfected with siRNA to specifically target PFN1 or negative control siRNA, and then the proliferation and metastasis of the transfected cells were measured. The transfection efficiency was confirmed by semiquantitative RT-PCR and Western blotting. The data showed that PFN1 expression in the siRNA-PFN1 group was reduced significantly at both the mRNA (24 h) and protein (48 h) levels when compared to the levels in the negative control siRNA and untreated control groups (Figure 5A). Next, the proliferation of siRNA-transfected Hep-2 cells was determined by CCK-8 assay. As shown in Figure 5B, within 48 hours, the percentages of viable cells were not significantly different in the PFN1 siRNA group when compared to the negative control siRNA and untreated control groups. And the cells of PFN1 siRNA group showed a significantly decreased proliferation at 72 h time points. Additionally, transwell assay was performed to further determine whether the downregulation of PFN1 could influence the migration ability of Hep-2 cells. As expectably, the numbers of cells in the siRNA-PFN1 group that migrated to the lower surfaces of the transwells were reduced in comparison to those in the negative control siRNA and untreated control groups (Figure 5C and D). These results indicate that PFN1 silencing affects the proliferation and migration ability of Hep-2 cells.

## Discussion

Previous proteomics study using 2D-MS identified the differential proteins in laryngeal carcinoma tissue [17], few candidate proteins were detectable because of low resolution, and most of the differentially expressed proteins detected were high-abundance proteins. In addition, using SELDI-TOF-MS method to identify the proteomic shift in laryngeal carcinoma serum, Cheng et al and Liu group reported different findings and conclusions that few proteins were found to vary in concert, and the discrepancies might be due to their technical problems such as varying ability of mass spectrometry to identify a particular protein [14], [15]. Thus, successful application of proteomic technologies to biomedical and clinical research is leading to the discovery of disease-specific biomarkers for diagnosis and treatment monitoring, providing insight into the underlying pathologies and allowing identification of novel therapeutic targets [12]. In the current study, we used 2 chromatographic methods coupled with MS to detect differentially expressed proteins and thereby greatly raised the number of detectable proteins. The 2D LC-MS/MS analysis performed in this study led to the identification of 1738 proteins, among which 281 were differentially expressed with significance between the laryngeal carcinoma tissues and the corresponding adjacent noncancerous tissues. Of these, 141 proteins were upregulated, and the remaining 140 proteins were downregulated. To get more insight on the biological significance of the differentially expressed proteins in laryngeal carcinoma process, hierarchical cluster, gene ontology and protein network analysis were performed on 281 differential proteins. Stage-specific and coregulated expression profiles of the differentially expressed proteins were displayed in the hierarchical cluster analysis. GO analysis revealed that each functional group may play a distinctive role during laryngeal carcinoma carcinogenesis. Additionally, the network diagram confirmed the relevance of the differentially expressed proteins provided a handle by which to identify upstream activators and downstream effectors.

Considerable proteins, such as YWHAZ, S100-A11, glutathione S-transferase, alpha-enolase, flavin reductase, fascin, and carbonic anhydrase, have been reported to be associated with laryngeal carcinoma in previous proteomics studies but without clinical validation and in-depth functional research [17], [18]. However, in our present study, four of the altered expressed proteins with different subcellular localization, such as PFN1: extracellular, NCL: nucleolus, nucleus and cytoplasm, CNDP2: cytoplasm, and OGN: extracellular, have been observed to be differentially expressed in cancers from other origins but not previously in laryngeal carcinoma [21]–[24]. Meanwhile, these candidates have been proved to be involved in multiple cellular pathways related to carcinogenesis, including proliferation, differentiation, apoptosis, migration, and invasion. Thus, the expression of PFN1, NCL, CNDP2 and OGN were further investigated employing a large collection of human laryngeal carcinoma tissues. Noteworthy, the effects of PFN1 in the proliferation and migration of human squamous cells were also analysed.

Profilin-1(PFN1), as an important actin-binding protein and ubiquitously expressed profilin isoform, has been considered as an essential control element for actin polymerization by virtue of its ability to funnel actin monomers (G-actin) to the growing filament and interact with almost all major protein families, which involved in nucleation and/or elongation of actin filaments [25]. Deregulation of PFN1 has been reported in various adenocarcinomas (breast, pancreas, hepatic, and gastric), and indicating that the molecule may function as a tumor-suppressor gene [26]–[29]. Especially, PFN1 plays crucial roles in metastasis and carcinogenesis of mammary epithelial cells by regulating membrane protrusion, motility, and invasion [30]. To know whether down-regulation of PFN1 is involved in laryngeal carcinoma carcinogenesis, we knocked down PFN1 in human laryngeal carcinoma cells Hep-2, and then detected whether PFN1 knockdown decreased the proliferation and metastasis of Hep-2 cells. The data showed that PFN1 silencing inhibited the proliferation and affected the migration ability of Hep-2 cells, demonstrating that PFN1 plays an important role in human laryngeal carcinoma carcinogenesis. To our knowledge, this is the first report to establish a correlation between PFN1 down-regulation and carcinogenesis of human laryngeal carcinoma, and PFN1 as a potential biomarker for early detection of this cancer.

Nucleolin (NCL) is another protein found overexpressed in laryngeal carcinoma. As a multifunctional phosphoprotein, NCL has a bipartite nuclear localization signal sequence and binds RNA through its RNA recognition motifs [31]. It has been shown to be up-regulated in highly proliferative cells and regulated many aspects of DNA and RNA metabolism, chromatin structure, rRNA maturation, cytokinesis, nucleogenesis, cell proliferation and growth [32], [33]. Further, the expression of NCL was reported to be increased in pancreatic ductal adenocarcinoma and the overexpression of the protein was found in other human cancers such as gliomas, melanoma, and non-small cell lung cancer [34]–[37]. Similarly, our semiquantitative RT-PCR and Western blotting results confirmed on a larger series of specimens the increased expression of NCL in the laryngeal carcinoma, indicating the possibility that the overexpression of this protein is more specific to cancer.

Cytosolic non-specific dipeptidase 2 (CNDP2), also known as carboxypeptidase of glutamate-like (CPGL), is expressed in all human tissues [38]. Previously, Zhang et al observed that CNDP2 is downregulated in hepatocellular cancer and could inhibit the viability, colony formation, and invasion of hepatocellular carcinoma cells [23]. A recent report also demonstrated that the loss of CNDP2 functioned as a tumour suppressor gene in pancreatic cancer and that the loss of CNDP2 suppressed proliferation, induced G0/G1 accumulation, and inhibited the migration ability of a pancreatic cancer cell line [39]. However, not all tumours express a low CNDP2 level, and the molecular function of CNDP2 is largely unknown. Okamura et al showed through quantitative proteomic analysis that renal cell carcinoma tissues have a high level of CNDP2 expression [40]. Tripathi et al found that CNDP2 was up-regulated in breast cancer tissues compared with normal breast epithelium [41]. The discrepant expression of CNDP2 in different tumours may due to its tissue specificity. In consistent with the later researches, our proteomic investigation revealed the overexpression of CNDP2 in the laryngeal carcinoma, and providing the information that this protein might be an accessible biomarker for certain type of cancers.

Mimecan (OGN), a secretory protein, belongs to a family of small leucine-rich proteoglycans (SLRPs). The expression of OGN was absent in several cancer cell lines, implicating its potential role as a tumor suppressor gene in cancer biology, although its physiological function has not been fully elucidated [42]. Even though, various human diseases, such as primary open-angle glaucoma and pituitary tumors, have been reported to associate with the expression of OGN [43]. Concomitantly, the differential expression of this protein serves as an excellent pathological biomarker to distinguish non-small cell lung cancers from small cell lung cancers [44]. Here, our validation experiments demonstrated the significant down-expression of OGN in a large group of laryngeal carcinoma patients, hinting that OGN may be a potential tumour suppressor gene involved in laryngeal carcinoma initiation and progression.

Taken together, in this study, the use of 2D LC-MS/MS identified 281 significantly differentially expressed proteins in human laryngeal carcinoma, and four differential proteins (PFN1, NCL, CNDP2 and OGN) with expressional changes were selectively verified. It was showed that panel of the four proteins, or some of them, could serve as novel potential biomarkers for detection or therapeutic targets of human laryngeal carcinoma. Moreover, it was found that PFN1 knockdown decreased the metastasis of Hep-2 cells, demonstrating that PFN1 plays an important role in metastasis of laryngeal carcinoma. Thus, our findings reported here could have potential clinical value in diagnosis of human laryngeal carcinoma, and would provide some valuable information for further study of molecular mechanisms of this cancer.
